# Fostering collaborative research for rare genetic disease: the example of niemann-pick type C disease

**DOI:** 10.1186/s13023-016-0540-x

**Published:** 2016-12-01

**Authors:** Steven U. Walkley, Cristin D. Davidson, Jonathan Jacoby, Philip D. Marella, Elizabeth A. Ottinger, Christopher P. Austin, Forbes D. Porter, Charles H. Vite, Daniel S. Ory

**Affiliations:** 1 Dominick P. Purpura Department of Neuroscience, Rose F. Kennedy Intellectual and Developmental Disabilities Research Center, Albert Einstein College of Medicine, 1410 Pelham Parkway South, Bronx, NY 10461 USA; 2 Hide and Seek Foundation for Lysosomal Disease Research, 6475 East Pacific Coast Highway, Suite 466, Long Beach, CA 90803 USA; 3 Dana’s Angels Research Trust, 15 East Putnam Ave., #117, Greenwich, CT 06830 USA; 4 Division of PreClinical Innovation, National Center for Advancing Translational Sciences, National Institutes of Health, Rockville, MD 20850 USA; 5 National Center for Advancing Translational Sciences, National Institutes of Health, Bethesda, MD 20817 USA; 6 Division of Translational Medicine, Eunice Kennedy Shriver National Institute of Child Health and Human Development, National Institutes of Health, DHHS, Rm 5-2571, 10CRC, 10 Center Dr, Bethesda, MD 20892 USA; 7 Department of Clinical Studies, School of Veterinary Medicine, University of Pennsylvania, 3800 Spruce Street, Philadelphia, PA 19104 USA; 8 Diabetic Cardiovascular Disease Center, Washington University School of Medicine, Box 8086, 660 S. Euclid Ave, St. Louis, MO 63110 USA

**Keywords:** Cyclodextrin, Collaborative science, Drug pipeline, Lysosomal disease, Miglustat, Niemann-Pick C, Patient advocacy, Rare disease, Therapy development, Translational medicine

## Abstract

Rare disease represents one of the most significant issues facing the medical community and health care providers worldwide, yet the majority of these disorders never emerge from their obscurity, drawing little attention from the medical community or the pharmaceutical industry. The challenge therefore is how best to mobilize rare disease stakeholders to enhance basic, translational and clinical research to advance understanding of pathogenesis and accelerate therapy development. Here we describe a rare, fatal brain disorder known as Niemann-Pick type C (NPC) and an innovative research collaborative known as Support of Accelerated Research for NPC (SOAR-NPC) which illustrates one pathway through which knowledge of a rare disease and its possible treatments are being successfully advanced. Use of the “SOAR” mechanism, we believe, offers a blueprint for similar advancement for many other rare disorders.

## Background

Although defined somewhat differently country to country, it is broadly estimated that there are approximately 7,000 rare diseases. In the U.S., a rare disease is defined as affecting fewer than 200,000 individuals, and rare diseases collectively as many as 30–35 million people. In Europe estimates are similar, with as many as 350 million individuals affected worldwide. Yet such large numbers stand in sharp contrast to the reality of most rare diseases for which there are only a handful of diagnosed individuals in an entire country. While the majority of rare diseases are genetic and the genes and mutations known for a rapidly increasing proportion, knowledge of disease often ends there, with only partial understanding of the role of the defective protein in cells and even less being known about disease pathogenesis. Nonetheless, understanding the function of rare disease-linked genes and their proteins can provide new insights into how cells function, which in turn may lead to insights into, and therapies for, both rare and common diseases.

Not surprisingly, the road from rare disease discovery to implementation of successful therapies is inevitably long and must take into consideration many factors. These range from disease recognition and discovery of the cause of the disease and its pathogenesis, to natural history studies to provide a clearer understanding of how a particular rare disorder affects different individuals, to establishment of clinics to facilitate patient care. For some, discovery of the disease may have been more than a century ago, as for diseases like Niemann-Pick, whereas for others a matter of only a few years or less. While much progress has been made in defining the molecular basis of many genetic diseases, large gaps remain in our understanding of the complex chain of events between the underlying genetic defect and the phenotypic abnormalities at various levels from cells, tissues, and organs, to the whole organism. Since studies necessary for unraveling these pathogenic mechanisms often cannot be done in humans or with human tissues, animals with the same genetic disorders are a critical resource for knowledge advancement. Appropriate animal models also provide a means to test new therapies, with mice, cats, dogs, and non-human primates increasingly recognized as important intermediate models to assess these strategies.

While a key goal for rare disease advancement is the development of clinical trials to test potential therapies, limited availability of affected patients often imposes significant constraints that require international cooperation to overcome. Fundamental to progress is the development of natural history studies, the purpose of which is to characterize the range of phenotypic heterogeneity and variability of disease progression. Information is obtained about frequency of signs and symptoms, age of onset, and disease progression. This information can be used to identify potential outcome measures for later therapeutic trials. Once the variability and rate of progression of a specific disease-related sign or symptom is known, the information can be used to adequately power a controlled clinical trial. From a natural history study, one also quickly learns information regarding the practicality and potential difficulties associated with obtaining data on a specific outcome measure. A second key aspect of natural history studies is that they provide a mechanism to collect patient biomaterials, including serum/plasma, urine, cerebrospinal fluid (CSF), DNA, and patient cells. These biomaterials provide a resource that can be used by scientists in studies focused on understanding the pathological processes underlying the disorder, as well as used to identify biomarkers to facilitate diagnosis or as tools to support the development of clinical trials. Such biomarkers may be any identified biochemical, genomic or proteomic feature, or even an imaging marker that serves as a measurable indicator of the disease state. Once validated in clinical trials against clinical outcome measures, these biomarkers have the potential to serve as surrogate outcome measures. Ultimately if validated as a surrogate, since they may respond quicker than clinical signs or symptoms, biomarkers could accelerate drug development by facilitating shorter and smaller clinical trials. Natural history studies are complemented by patient registries, which serve as a resource to identify patients who may be interested in participating in a clinical trial as well as to provide information on disease phenotype and progression. They have the potential advantage of being able to ascertain information from individuals who are not able to travel to a study site in order to participate in a natural history study.

Development of natural history studies and registries, biomarker discovery, and expanded understanding of disease pathogenesis through availability of animal models (that can also be used for preclinical testing of specific treatments), effectively set the stage for clinical trials. Formal clinical trials in which data are collected in a rigorous and standardized manner are essential for approval of a drug by regulatory agencies. Prior to approval of a drug the Food and Drug Administration (FDA) requires that efficacy be shown “through adequate and well-controlled clinical studies” (21CFR314.126). Although well-documented case reports and case-series may provide information related to safety and insight into potential outcome measures for a subsequent well-controlled trial, they are not controlled studies and therefore would not be adequate for regulatory approval.

**Fig. 1:**
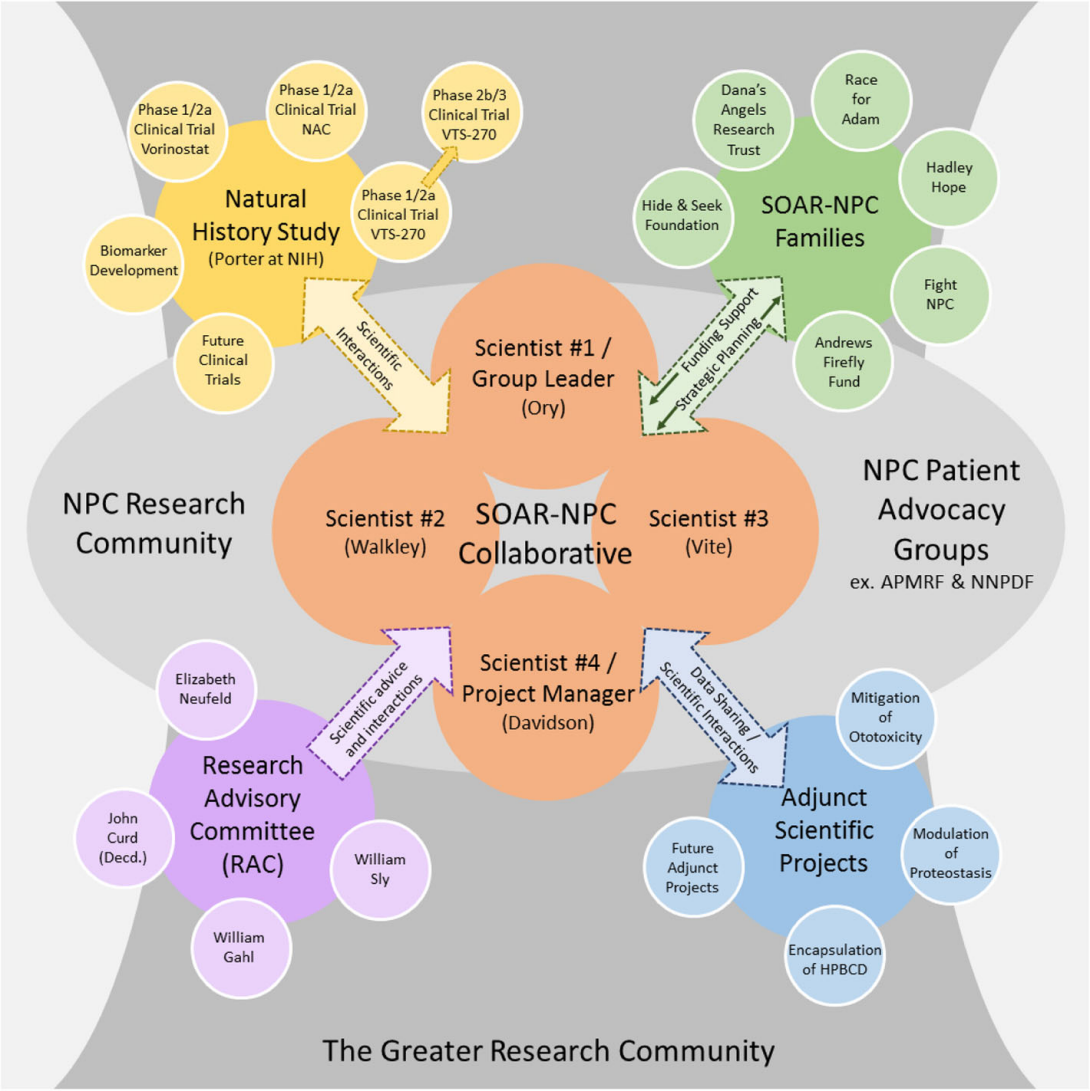
SOAR-NPC organizational chart. At the center of SOAR-NPC is the Collaborative, the scientific core which maintains vital relationships and open communication with other key groups (*arrows*). Ongoing interactions amongst groups facilitate SOAR-NPC’s ability to rapidly respond to a dynamic research environment. See text for additional details

While the above cycle of progress for a rare disease may appear fully exploitable through an academic medical center or network of such centers, in reality, the importance of patient advocacy as catalyst and supporter of these studies cannot be overstated. Disease advocates participate and have direct input in the process by bringing scientists together, organizing within the patient community, holding meetings/conferences with scientists and patient advocates, and funding early-stage, high-risk research through grants, both directly to researchers and by leveraging other funding. It is also critical to foster and support public-private partnerships by developing relationships with government agencies and industry. Patient liaisons that learn and understand the drug development and FDA regulatory process empower advocates to be more fully engaged and to interact with scientists and regulators. In order to build a collaboration and strong partnership, it is important to establish a relationship of trust and engagement between patient advocates and the scientists/clinicians to foster productive ways of working together. Key elements of the relationship are communication and mutual respect for each other’s roles, including the establishment of defined boundaries and organizational structure so that the relationship remains collaborative and productive. Scientists know science and are pragmatic; parents are pragmatic in a different way, focused on saving their children’s lives and bringing other perspectives and strategies to solving problems and implementing ideas that benefit the patient community. Several years ago a group of scientists, clinicians and parents came together in an attempt to overcome the obstacles inherent in developing an effective therapy for the rare brain disease known as Niemann-Pick type C, and in the process created an innovative research collaborative known as Support of Accelerated Research for Niemann-Pick C disease (SOAR-NPC) (Fig. 1). That experience is described here and we propose that it can serve as a basis for advancing therapies for other rare disorders.

### Niemann-pick type C disease, a model example

NPC disease is a rare, progressive, neurodegenerative disorder characterized by storage of unesterified cholesterol in the late endosomal/lysosomal system. Two genes (*NPC1* and *NPC2*) have been linked to NPC disease in humans, with 95% of cases associated with mutations in *NPC1*. NPC1 is a multi-spanning transmembrane protein and NPC2 is a soluble cholesterol-binding protein, both of which reside in late endosomes/lysosomes [1]. The incidence of classical NPC1 disease has been estimated to be 1/89,000-104,000 [1], whereas a late-onset variant of NPC1 has a predicted incidence of 1/39,000 based on analysis of *NPC1* carrier frequency from large-scale exome sequencing [2]. Affected individuals typically present with visceral involvement as neonates, progressing in early childhood with ataxia and impairment of motor and intellectual function, and usually die in adolescence. Late-onset NPC1 disease increasingly is being recognized in young adults with psychoses and cognitive impairment, with a disease prevalence of ~2% among this population [3].

The discovery of NPC1 disease biomarkers has transformed the diagnosis of NPC1 disease and accelerated drug development efforts. Until recently, the first-line diagnostic test for NPC disease was filipin staining of cultured primary skin fibroblasts for cholesterol. The discovery in one of the SOAR-NPC labs (Ory) that oxysterols are sensitive and specific disease markers [4] has led to development of a clinical assay that has been implemented in more than 30 laboratories worldwide and is replacing the filipin test as the diagnostic standard [5]. More recently, metabolomic profiling led to identification of unusual bile acid species that are specifically elevated in the plasma of NPC1 disease patients [6]. A high-throughput, mass spectrometry-based method was developed and validated to measure the bile acid biomarkers in dried blood spots with their quantification providing the basis for a newborn screen for NPC disease. This screen is now ready for piloting in newborn screening programs.

Establishment of an NPC disease natural history study at NIH in 2006 (NCT00344331) provided the means to characterize the clinical phenotype and phenotypic heterogeneity of NPC1 disease across a large number of patients in both a cross-sectional and longitudinal manner (Porter). The longitudinal nature of this study allowed for disease progression to be characterized and a Neurological Severity Score to be developed [7]. This scoring system assessed disease progression in nine major and eight minor sign/symptom domains that are clinically relevant to NPC disease progression. The scale was found to be useful both retrospectively and prospectively to characterize disease status and progression. The NPC1 disease natural history study also provided an opportunity to establish a biorepository of blood, CSF, and fibroblasts from well characterized patients. These samples subsequently proved invaluable in the identification and characterization of pharmacodynamic biomarkers [8] and biomarkers of neuronal damage such as tau [9], FABP3 [10] and calbindin D [11]. These biomaterials were also essential for development of the assay for newborn screening for NPC disease mentioned earlier [6].

In addition to defining the gene defect in NPC1 disease, developing disease-specific biomarkers and establishing a natural history study, the NPC research community has also identified disease models in multiple species. These include simple non-mammalian systems – *S. cerevisiae*, *C. elegans*, *D. melanogaster*, and *Danio rerio-*as well as *M. musculus* and *F. catus* mammalian models. Several mouse models are available including both spontaneous mutations (e.g. BALB/cNctr-*Npc1* ^*m1N*^/J, C57BLKS/J-*Npc1* ^*spm*^/J) and targeted or chemically induced mutations (e.g. B6.129-*Npc1* ^*tm1.1Dso*^/J, p.I1061T, c.3182 T > C; C57BL/6 J-*Npc1* ^*nmf164*^/J, p.D1005G, c.3163A > G; and an NPC2 hypomorphic allele). The most widely used mouse model is the *Npc1* ^*m1N*^, a null model initially characterized at the NIH [12–14]. These mice exhibit weight loss, severe ataxia, and intracellular accumulation of cholesterol and other lipids, all prominent clinical and pathological manifestations seen in NPC1 patients. A humanized hypomorphic *Npc1* ^*tm1.1Dso*^ mouse model was also recently developed in one of the SOAR-NPC labs (Ory). Here, the p.I1061T (c.3182 T > C) mutation, the most prevalent human disease allele, was inserted into the *Npc1* locus [15]. *Npc1* ^*I1061T/I1061T*^ “knock-in” mice show an increased mean survival time as compared with *Npc1* ^*m1N/m1N*^ mice, consistent with partial function of the mutant protein. Onset of neurological signs is delayed 3 weeks, and the progression of signs and weight loss are less precipitous.

The discovery of a large animal model of NPC1 disease occurring spontaneously in cats [16] has proven to be a critical resource for advancing therapy. Here, the disorder results from a single missense mutation in *NPC1* (p.C955S, c.2864G > C) that most closely models the clinical disease features of juvenile-onset NPC1 disease patients (the most common form of NPC1 disease) [17]. The cat model displays ectopic dendritogenesis, a key neuropathological feature of many lysosomal disorders in humans and is a finding that occurs only minimally in murine models of these diseases [18]. Furthermore, the NPC1 cats exhibit a massive degree of neuroaxonal dystrophy, thereby recapitulating another critical aspect of the cellular pathology of NPC1 disease. A breeding colony of NPC1 cats has been established at the National Referral Center for Animal Models of Human Genetic Disease of the School of Veterinary Medicine of the University of Pennsylvania by one of the SOAR-NPC scientists (Vite). The size of the feline model allows for the ability to both repeatedly administer experimental therapies intravenously or intrathecally and to repeatedly sample blood, urine, and CSF. This model has also allowed for validation of biochemical markers of disease severity and therapeutic effects that are specific to central nervous system (CNS) disease. Finally, its large size also allows for the development of metrics of disease progression using equipment identical to those used in people, e.g., magnetic resonance imaging (MRI) and electrodiagnostic testing. The cat model has been extensively used to evaluate the efficacy of experimental therapies of NPC disease [19–21].

The mammalian models of NPC1 disease have been particularly valuable in allowing for studies of NPC disease pathogenesis. Through such analysis it is now known that cholesterol, glycosphingolipids (GSLs), particularly GM2 and GM3 gangliosides and lactosylceramide, are abnormally sequestered in brain cells in NPC disease [22, 23]. Endosomal/lysosomal storage of gangliosides without clear evidence of deficiency of ganglioside catabolic enzymes in the NPC1 mouse and cat models led Walkley and colleagues to speculate that this might be the result of increased GSL synthesis [24]. Subsequent studies using the GSL synthesis inhibitor, N-butyl-deoxynojirimycin or miglustat, in the NPC1 models were the first to demonstrate delay in onset of neurological disease and increased longevity for this disease [25], a finding subsequently extended with additional studies in the feline model [19]. Today, miglustat is an approved therapy for NPC1 disease in 45 countries, though not in the U.S.

Most of the support for the above studies on NPC disease was derived from work funded through the “classic” model of peer-reviewed investigator-initiated projects. These were largely funded through the NIH using the widely recognized “R01” and “R21” mechanisms and by private foundations such as the Ara Parseghian Medical Research Foundation (APMRF) and the National Niemann-Pick Disease Foundation (NNPDF) following review by their scientific advisory boards. Importantly, funding by these private foundations often provided critical support for high risk, high impact projects that would not have been supported via traditional funding routes, such as the NIH. Grants supported by the APMRF, for example, led directly to identification of the NPC1 gene, to improved understanding of the function of the NPC1 and NPC2 proteins and to the discovery of miglustat’s ameliorative effects on NPC disease. The APMRF also provided critical support for the initiation of the NPC natural history study described earlier. Yet despite these and other important advances for NPC disease, there was also a growing realization among some parents that a critical element for progress was missing. This was the pre-clinical and clinical expertise similar to that which pharmaceutical companies would have for effectively and fully developing therapies for this fatal neurodegenerative disease. It was in this mixed atmosphere of hope and realization that a complementary approach to research support was born, one focused specifically on development of NPC1 disease therapies.

### Emergence of SOAR-NPC

The idea of the research collaborative known as SOAR-NPC emerged when three researchers and a parent traveled to Brazil in 2007 to visit the family of a young adult patient who had the disease. The patient’s father was a potential funder of NPC disease research and the visitors wanted to prepare something more than a simple listing of potential therapeutic strategies. Inspired by a collaborative research model that had been developed by the Myelin Repair Foundation [26], SOAR-NPC was conceived to incentivize and maximize scientific collaboration. Its ambitious objective, stated at the outset, was to develop, within 3–5 years, an FDA sanctioned clinical trial with emphasis on development of a combination therapy-or “cocktail” – that would prevent or significantly delay the onset and progression of clinical neurological disease in individuals affected by this disorder. Toward this end, the initial family foundations in support of SOAR-NPC (Dana’s Angels Research Trust and the Hide and Seek Foundation for Lysosomal Disease Research) established a funding stream for NPC disease research in 2008 prior to its first multi-party organizational meeting at Stanford University.

In creating the SOAR-NPC Collaborative (Fig. 1), the researchers and families early on embraced the concept of a small network of highly interactive/collaborative scientists. Such individuals, it was decided, need not be at the same institution or even in the same country; rather, it was critical to have a detailed knowledge and involvement with NPC disease, and equally important, a strong willingness by each scientist to be truly collaborative, planning experiments and sharing data collection and analysis in real-time. It was decided that scientists entering the SOAR-NPC Collaborative must be willing to share unpublished data to aid in rapid and efficient progress. Hosting conference calls every other week was viewed as an essential element in coordinating activities. The number of scientists was also thought to be critical. Too many, and the close coordination between labs might be lost, yet too few and a deficiency in critical complementary skills might weaken research progress. Since the establishment of SOAR-NPC the number of scientists has ranged from 3 to 4, with 2 of the scientists (Ory and Walkley) being part of the collaborative since its inception. While the SOAR-NPC scientists elected their own group leader (Ory), it was also quickly realized that the group would need a scientific coordinator to act as project manager as well as liaison with the SOAR-NPC parent groups. This role was filled by different individuals over the years (including Ottinger) and continues to evolve with the changing needs of SOAR-NPC. Currently, this individual (Davidson) schedules the biweekly conference calls and biannual face-to-face meetings of the scientists and parents, as well as follows up on research questions arising in the scientific exchanges during the teleconferences or in discussions with parents. As unpublished data is constantly under discussion, agreement to maintain confidentiality by all parties involved has proved vital to success. Given the importance of the goal to accelerate therapy for NPC1 disease, the collaborative established connections early on to the NPC1 disease natural history study being carried out at the National Institutes of Health (Porter). With the discovery of 2-hydroxypropyl-β-cyclodextrin’s (HPβCD) remarkable efficacy in animal models, this connection proved essential for rapid acceleration toward a clinical trial through links with NIH’s Therapeutics for Rare and Neglected Diseases (TRND) Program in the new National Center for Advancement of Translational Science (NCATS), as described below. Critical to the operation of SOAR-NPC has been its ability to identify and fund investigators outside the SOAR-NPC Collaborative who are pursuing projects directed at therapy for NPC1 disease. These adjunct projects, coordinated by the SOAR-NPC research manager, have varied widely from those focused on proteostatic and gene therapies, to preventing ototoxicity following administration of cyclodextrin, to developing improved HPβCD formulations that cross the blood–brain barrier. Such pilot projects typically have lasted a year, or more, with funded scientists joining biweekly calls with the SOAR-NPC scientists as data was ready for analysis and discussion (Fig. 1).

**Fig. 2:**
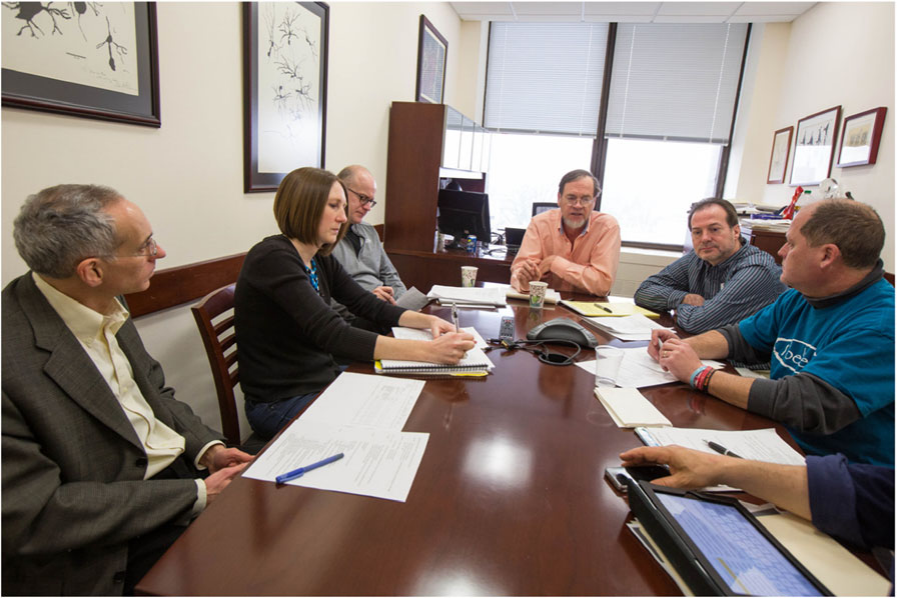
SOAR-NPC Collaborative meeting. A face-to-face meeting of SOAR-NPC members as occurred at the Rose F. Kennedy Center at the Albert Einstein College of Medicine on February 26, 2014. Pictured are (*left* to *right*), Daniel S. Ory, Cristin D. Davidson, Charles H. Vite, Steven U. Walkley, Philip D. Marella and Sean Recke (not shown: Jonathan Jacoby). Individuals shown have consented to having their pictures published

The collaborative character of SOAR-NPC is not limited to its scientific members as parents/guardians also play a key role in a number of ways. The SOAR-NPC families presently represent six individual foundations, each of which pursues their own avenue(s) to raise money to support NPC disease research. Resources are then pooled in order to partially fund not only the SOAR-NPC scientists, but also the smaller adjunct projects mentioned above that align with SOAR-NPC goals. Biannual face-to-face meetings (Fig. 2) between SOAR-NPC families and its scientists serve to not only share progress of studies, but importantly, to reach consensus on what avenues are worth pursuing in future research efforts, including those small pilot projects outside of main SOAR-NPC laboratories. Whenever necessary as part of such decisions on current and future research directions, the SOAR-NPC Collaborative seeks advice from its Research Advisory Committee (RAC). This group, which was used most often in the early years of SOAR-NPC, represents a small group of senior investigators in the lysosomal disease and drug development fields (Fig. 1). Overall, the biannual meetings of the Collaborative not only allow progress and plans to be reviewed, but also provide an opportunity for the SOAR-NPC families to understand the scientific merit of proposed projects, permitting them to make informed decisions on how pooled resources are utilized.

Beyond raising significant funds to support NPC1 disease research, parents are also involved in developing intellectual property agreements as well as fostering new relationships with researchers and the pharmaceutical industry, in representing the NPC1 disease community with government agencies, and in facilitating more regular and effective communication among the various stakeholders. Perhaps most importantly, parents bring a practical sense of urgency to the therapy-development project. Just as they learn the realities and practicalities of scientific research, parents bring their skill sets and personal experiences to the SOAR-NPC enterprise in a way that has tangibly accelerated the development of therapeutic strategies.

The other major stakeholder in SOAR-NPC has been the NIH, which has a long history as a partner in the basic research and development of therapeutics for NPC1 disease and as a facilitator of interactions between the scientific and the NPC1 disease parent and patient communities. Scientists at NIH first identified the cellular defect in 1985 [27] followed years later by the cloning of the *NPC1* gene in 1997 [28] and subsequently implemented the first clinical trial to test cholesterol-lowering drugs. Shortly after SOAR-NPC was established in 2007, its scientists met with researchers at the NIH Chemical Genomics Center (NCGC), including its then scientific director, Dr. Christopher P. Austin, in order to discuss screening of patients’ cells, provided by participants enrolled in the on-going NPC disease natural history study at NIH, with a collection of FDA-approved drugs. The goal was to look for molecules that could reverse cholesterol storage in cells, and as a result be “repurposed” as a therapy for NPC1 disease, potentially shaving years off the drug development timeline. In 2008, the NCGC received a grant from the APMRF to support this high-throughput screening effort. The collaboration with SOAR-NPC investigators involved testing the best lead compounds identified from the screening effort for *in vivo* efficacy in the NPC disease animal models. The initial lead compound identified from this screen and subsequently tested in the NPC1 (BALB/cNctr-*Npc1* ^*m1N*^/J) mouse model was a component of Vitamin E [29], but the compound failed to show efficacy in the mouse. Nonetheless, the close working relationship established between the NCGC and SOAR-NPC scientists on this project laid the foundation for cooperative development of a new lead compound that was just emerging from several laboratories working on NPC1 disease.

### The discovery of cyclodextrin as therapy for NPC disease

By the early 2000s, an improved understanding of NPC1 disease allowed scientists to focus more efforts on rational therapeutic approaches, such as targeting downstream effects, reducing accumulating metabolites, and as applicable, enhancing existing protein function. Projects focused on therapeutic intervention became more commonplace. Around the same time the idea of SOAR-NPC was taking shape, exciting new data from two independent laboratories, John Dietschy, a scientist at University of Texas Southwestern and Steven Walkley (a SOAR-NPC scientist) was being shared at the 2007 APMRF annual meeting. Here, both investigators reported the unexpected and surprising realization that HPβCD, the vehicle or “excipient” used to solubilize a putative therapeutic compound (allopregnanolone) [30], provided far greater benefit in the NPC1 mouse model than allopregnanolone itself, or indeed any compound investigated thus far, including miglustat. Though an earlier study investigated HPβCD for treatment of NPC1 disease, use of a much lower dose failed to elicit the significant responses subsequently reported by the Dietschy and Walkley labs [31–34]. Importantly, the studies from these two groups revealed that treated mice not only exhibited delayed onset of clinical disease, but also showed dramatically less storage of cholesterol and sphingolipids and lived nearly twice their normal lifespan. Subsequent studies in a second SOAR-NPC lab (Vite) treating the feline NPC1 model with HPβCD further advanced the notion of this compound’s ability to significantly impact the disease process. Indeed, direct administration of HPβCD into the cerebellomedullary cistern of presymptomatic cats with NPC disease prevented the onset of cerebellar dysfunction for greater than a year, and resulted in Purkinje cell survival and near normal concentrations of brain cholesterol and sphingolipids [21]. At the same time, an adverse effect identified was an increase in hearing threshold. Together, these studies in the feline model provided critical data on efficacy and relative safety of direct CNS administration of the drug that have proven important in advancing the subsequent clinical trials. These substantial preclinical studies, combined with HPβCD's overall safety profile and many years of experience as an excipient in FDA-approved drugs, were largely responsible for promoting this compound as SOAR-NPC’s lead candidate for advancement to clinical trial. During the latter period of preclinical research, several NPC1 disease families received approval from the FDA for individual expanded access Investigational New Drug (iIND) applications and their children began receiving HPβCD intravenously. While acknowledging the potential contribution of iINDs and first-in-human treatment with the drug for contributing information related to the safety of HPβCD, numerous investigators involved in HPβCD preclinical work and patient advocates realized the critical importance of carrying out a scientifically rigorous clinical trial to unambiguously establish safety and potential efficacy of this compound, so that it could eventually gain FDA approval. Accordingly, this effort became the common goal amongst all involved in the push towards establishing HPβCD as a bona fide therapy for NPC1 disease.

In 2010, the NIH established the aforementioned program known as TRND which subsequently became an integral component of NCATS and incorporated the NCGC scientists with whom SOAR-NPC had previously collaborated. TRND was specifically designed to stimulate drug discovery and development for rare and neglected diseases through a collaborative model between the NIH, academic scientists, nonprofit organizations, and pharmaceutical and biotechnology companies. That same year, the National Institute for Neurological Disorders and Stroke (NINDS) sponsored the meeting, “Promising Therapies for NPC,” organized by Drs. Steven Walkley and Danilo Tagle, to bring together NPC1 disease researchers and disease advocacy groups at NIH to discuss the current state of knowledge and possible therapeutic developments, focusing in part on the growing awareness of the promise of HPβCD for treating NPC. SOAR-NPC quickly recognized the potential for the TRND program to provide critical support for development of this compound as a therapeutic drug. In collaboration with Dr. Forbes Porter at the NIH, SOAR-NPC scientists (Ory and Walkley) developed a research proposal that provided a roadmap for rapid translation of preclinical studies of HPβCD to a Phase 1/2a trial at the NIH Clinical Center. The proposal was accepted and in early 2011 development of HPβCD for the treatment of NPC1 was selected as one of TRND’s initial projects [35].

**Fig. 3:**
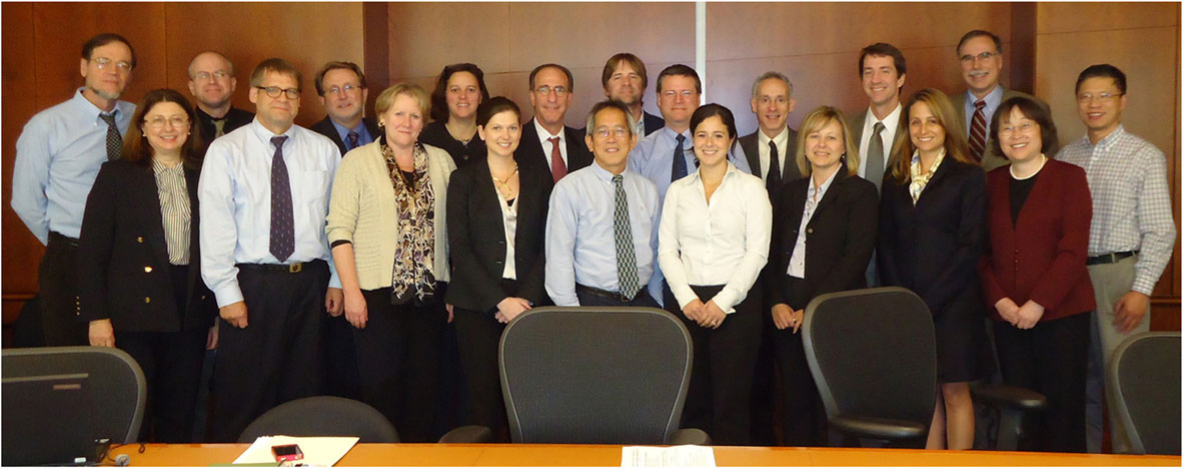
TRND-NPC team members. First Row (*Left* to *Right*): Ilona Scott (Janssen Research & Development, LLC, Johnson & Johnson [J&J]), Patrick Frenchick (RRD International, Inc. [RRD]), Sandra L. Morseth (RRD), Kimberly Lilly (RRD), Mark L. Kao (J&J), Nicole Y. Farhat (NIH/NICHD), Elizabeth A. Ottinger (NIH/TRND), Nuria Carrillo (NIH/TRND), Xin Xu (NIH/TRND); Second Row (*Left* to *Right*): Steven U. Walkley (Albert Einstein College of Medicine), Charles H. Vite (University of Pennsylvania), Charles Finn (RRD), Joy K. Vander Wal (RRD), Steven A. Silber (J&J), John C. McKew (NIH/TRND), Forbes D. Porter (NIH/NICHD), Daniel S. Ory (Washington University School of Medicine), Christopher P. Austin (NIH/TRND), John Heiss (NIH/NINDS), Wei Zheng (NIH/TRND); Missing Team Members: Juan J. Marugan (NIH/TRND), William J. Pavan (NIH/NHGRI), James Cradock (NIH/TRND) and Pramod Terse (NIH/TRND) Individuals shown have consented to having their pictures published

This effort required the establishment of a large collaborative team with a wide range of expertise in all areas of drug development, including chemistry and manufacturing, formulation, pharmacology, pharmacokinetics, toxicology, and regulatory affairs, to advance the molecule from basic discovery to clinical testing. SOAR-NPC researchers (Ory, Vite and Walkley) became key members in establishment of the TRND-NPC team (Fig. 3), adding critical expertise in NPC1 disease animal models and biomarker discovery and assay development. In addition, a SOAR-NPC collaborator and leader of the NPC1 disease Natural History study at the NIH Clinical Center (Porter) provided clinical expertise in human NPC1 disease and patient care. Importantly, the TRND-NPC project manager (Ottinger) also provided project management for SOAR-NPC, serving as a liaison that helped to align the goals and interests of the parents with the researchers and their plan for developing a therapy for NPC1 disease. This centralized project management support and point of contact was found to be key to driving the project forward to meet milestones and the end goal, a Phase 1/2a clinical trial. It also ensured that the necessary resources were available, tracked the TRND-NPC team activities and studies, monitored overall project costs and fostered a cohesive team environment. This coordinated team effort facilitated completion of all the needed pre-clinical studies, leading to the successful initiation of the Phase 1/2a trial of a defined HPβCD preparation at the NIH Clinical Center in early 2013 [35, 36]. After years of effort in supporting NPC1 disease research and the close partnerships forged between advocacy groups and the research community, including the SOAR-NPC model, the NPC1 community had a drug candidate entering clinical studies. The extensive preclinical data package and early clinical data from the Phase 1/2a study, coupled with successful designation of HPβCD as an Orphan Drug in the U.S. and EU, sufficiently de-risked the HPβCD program such that TRND-NPC was able to able to attract an industry partner. Vtesse, Inc., a pharmaceutical company, was founded to develop a specific HPβCD preparation (VTS-270) for treatment of NPC1 disease, and the Phase 2b/3 international, multi-site trial was launched in September 2015. Vtesse’s VTS-270 recently received the FDA’s Breakthrough Therapy Designation, which could accelerate approval to provide the first effective treatment for slowing progress or stabilizing the devastating impacts of NPC in children and adolescents. The clinical trial has entered Phase 3 and once again the NPC community, including SOAR-NPC families and scientists, is mobilizing its efforts with a parent/patient clinical trial recruitment campaign called “51 and Done!” which acknowledges the number of patients to be recruited for the trial.

### SOAR-NPC – An evolving strategy

At its inception, SOAR-NPC’s primary goal was to initiate a clinical trial for NPC disease within a 3–5 year timeframe. Remarkably, this goal was accomplished in 2013, just under 5 years after the initiation of funding for the SOAR-NPC scientists. This success did not diminish the enthusiasm or desire to continue pursuit of different therapeutic compounds. Although VTS-270 holds substantial promise for treating NPC disease, SOAR-NPC acknowledges VTS-270’s limitations and the necessity of finding additive, and even more optimal and tractable therapies. Early on, the members of SOAR-NPC decided a drug pipeline was essential to the success of efficient compound evaluation. This drug pipeline prioritizes compounds of interest while maintaining flexibility. When the pipeline was first conceived in 2007, 12 compounds of interest were selected for evaluation. To date more than 30 potential drugs have been processed through this pipeline. While VTS-270 continues to receive scientists’ attention, greater emphasis has recently been placed on cutting-edge technologies such as gene therapy and modulators of the proteostatic network. Potential therapies failing to show improvements in *in vivo* studies are accordingly removed from the pipeline queue, thus allowing evolution and incorporation of new compounds brought to light through diligent awareness of new scientific literature by the SOAR-NPC scientists and by parents in the NPC1 disease community. As the list of compounds grows, so too does the number of researchers brought in to explore these different avenues. For example, a workshop jointly funded by SOAR-NPC and the APMRF on the topic of proteostatic modulation in NPC disease brought an additional five researchers into the fold as affiliate labs of SOAR-NPC. Efforts such as this workshop not only foster the growth of the NPC research community, but, equally as important, also increase its intellectual capacity and breadth.

## Conclusions

Today’s scientific environment is slowly transitioning from an atmosphere of scientists wary of sharing data before publication to one with a much greater appreciation for the power of collaboration. New relationships between academia, industry, government, and patient advocacy groups have allowed rapid progress and brought patient welfare to the forefront. Rare diseases, though often comprised of very small patient groups, are finding their way to the leading edge of research, sometimes for the simple reason that scientists find the missing gene and defective protein as a key to understanding some aspect of cell biology they are pursuing. Not only are scientists beginning to appreciate the applicability of knowledge gleaned from rare diseases to more common maladies, but the avenues and compounds being employed to treat these often lethal conditions are also pushing the therapeutic envelope. We have highlighted NPC1 disease and SOAR-NPC as an example of a successful pathway for increasing knowledge of a rare disease while efficiently advancing potential therapeutics to clinical trial. Certainly SOAR-NPC is not unique as an organization designed to advance rare disease research and therapy for lysosomal diseases, e.g., see Jonah’s Just Begun http://jonahsjustbegun.org/aboutus/; The ML4 Foundation, http://ml4.org/; Team Sanfilippo, http://teamsanfilippo.org/; Beyond Batten Disease http://beyondbatten.org/, the National Tay-Sachs and Allied Diseases Foundation, http://www.tay-sachs.org/and others. Yet, SOAR-NPC appears unique in its internal organization, which has been honed through incorporation of a number of key features (Table 1) to maximize its likelihood for success: selection of scientists willing to openly share data; incorporation of family foundations able to provide critical financial resources and who appreciate that the selected scientists both acknowledge the importance of and actively strive for therapy advancement; maintenance of flexibility from key players permitting adaptation to a rapidly changing environment, and welcoming the expertise of outside scientists, even if only for selected projects or a limited time; and most importantly, creation of a cohesive group of people with a shared vision to increase knowledge while simultaneously advancing therapeutics. Though there is no single correct formula for achieving success, we hope this blueprint using the “SOAR-NPC” model may help others in the rare disease community increase their knowledge and ultimately, improve the well-being of the nearly 350 million individuals worldwide who are united by the common designation of having a “rare” disease.

**Table 1:** How to establish a rare disease SOAR collaborative

1. Find your fellow patients, families, and patient advocates who want to accelerate therapy-oriented research and development for the disease in question. Important characteristics: ability to give/raise research funds; good communication skills; willingness to devote time; openness to new ideas; ability to maintain confidentiality when needed within the SOAR.
2. Interview and select a small group of scientists/physician scientists appropriate to the SOAR’s objectives. Selection should be based on their research backgrounds, their genuine willingness to work collaboratively and openly share unpublished data and their familiarity and willingness to establish relationships with patients and families.
3. Clearly identify and state main objectives of the SOAR. Be as specific as practical with regard to time frame, research strategy and/or development of treatment strategies.
4. Establish a coordinated system of collaboration and communication between the scientists, ideally with one scientist acting as the Chair of the scientific working group.
5. Identify a SOAR Project Manager to monitor and coordinate the scientific collaborative and to link the activities of the scientists with the patient advocates.
6. Schedule regular briefings for SOAR patient advocates and twice-annual in-person joint meetings of patient advocates/families with SOAR scientists.
7. Identify a small group of senior scientists to serve as an outside advisory group to be called upon as needed for advice.
8. Develop payment plans, grant agreements and milestones for researchers.

## Abbreviations

APMRF: Ara Parseghian medical research foundation
CNS: Central nervous system
CRADA: Cooperative research and development agreement
CSF: Cerebrospinal fluid
FDA: Food and drug administration
GSLs: Glycosphingolipids
HPβCD: 2-hydroxypropyl-β-cyclodextrin
iIND: Individual expanded access investigational new drug
NCATS: National center for advancement of translational science
NCGC: National chemical genomics center
NICHD: National institute of child health and human development
NIH: National institutes of health
NINDS: National institute of neurological disorders and stroke
NNPDF: National Niemann-pick disease foundation
NPC: Niemann-pick type C disease
NPC1: Niemann-pick C protein 1
NPC2: Niemann-pick C protein 2
RAC: Research advisory committee
TRND: Therapeutics for rare and neglected diseases
VTS: Vtesse
